# Alumina nanoparticle-assisted enzyme refolding: A versatile methodology for proteins renaturation

**DOI:** 10.1038/s41598-017-01436-6

**Published:** 2017-05-03

**Authors:** Katerina V. Volodina, David Avnir, Vladimir V. Vinogradov

**Affiliations:** 1 ITMO University, Laboratory of Solution Chemistry of Advanced Materials and Technologies, Lomonosova St. 9, 191002 St. Petersburg, Russian Federation; 2 Institute of Chemistry and the Center for Nanoscience and Nanotechnology, the Hebrew University of Jerusalem, Jerusalem, 9190402 Israel

## Abstract

We present a high-yield method for the renaturation of negatively charged enzymes. The approach is based on the use of alumina nanoparticles, which after electrostatic interaction with denatured protein molecules, prevent their aggregation and make the process of refolding controllable. The method, demonstrated by the renaturation of several enzymes, is efficient, rapid, employs a minimal amount of reagents and even can be applied to renature mixture of the denatured enzymes.

## Introduction

Proteins are important components in many drugs and food products and in the technologies of their production, and thus, their processing and stabilization in solutions has been extensively studied^1, 2^. In water, colloidal or interfacial assemblies with surfactants or nanoparticles provide conditions to control protein stability and folding/unfolding rearrangements and renaturation^3^. Several methods based on interface interactions for renaturation of proteins are available, which in general can be divided into three main groups: (I) methods to remove the denaturants from the protein solution^4–6^; (II) methods involving the control of the physical conditions used during the refolding process^7, 8^; and (III) methods in which refolding agents are added during the renaturing processes^9–11^. A major drawback of these methods has been the high specificity to a particular protein which requires tailoring of conditions in each case; from that point of view, a versatile chemical general tool, applicable for any target protein is yet to be developed, and is highly needed.

Among the most promising methods for the renaturation of proteins are adsorption methods, where the denaturants are washed away from adsorbed denatured proteins on solid matrices, allowing these biomolecules to subsequently refold upon release from these supports^12^. A variety of chromatographic supports, zeolites and other solid phases have been used for that purpose^13–20^. Yet, there are some drawbacks of the use of these solid-support methods, which hamper the effectiveness of this approach: The adsorption process and the subsequent desorption processes are prolonged and time-consuming; the adsorption/desorption processes are incomplete; there is high sensitivity of the renaturing process to protein concentration; and high volume of renaturing buffer solutions are needed in order to get reasonable yields.

Special role in the renaturation methods plays nanoparticles called synthetic or artificial chaperons^21–24^. Despite on high perspectives of these systems, they still have some drawbacks such as the possibility to renature only partially denatured enzymes^21^, the requirement of additional chemicals for increasing yield^22^ and high specificity^23, 24^.

**Figure 1:**
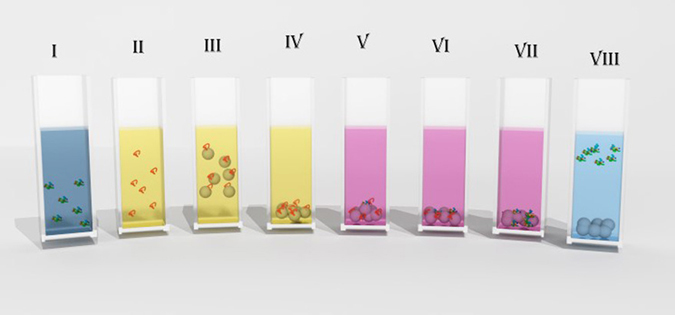
The alumina nanoparticle-assisted enzyme refolding process; see text for explanation of the steps.

In a previous report^25^ we described an alumina sol-gel assisted route for renaturing denatured enzymes. Remarkably, thermally denatured carbonic anhydrase was renatured to even higher activity (180%) compared to the free enzyme in solution. This “Phoenix” phenomenon was explained by a more favorable orientation of the enzyme within the alumina cages where the renaturation takes place. This observation, as well as the accumulated know-how of the use of solid-support adsorption methods described above, has led us to the development of an alumina nanoparticle-assisted renaturation tool, which is simple, of high-yield, and, even more important, versatile for many proteins. Here we report achieving this goal with a new concept, the steps of which - illustrated in Fig. 1 – are the following: After chemically denaturing the native enzyme (stages I and II), aggregation is prevented by adding the highly positively charged alumina nanoparticles (+45 mV), which, through their electrostatic binding affect the de-aggregation of the enzyme molecules (stage III). The resulting enzyme-alumina complexes can be easily centrifuged (stage IV) and separated from the denaturant by transferring to another buffer solution (stage V). Next, the denaturant is removed by several wash cycles (stage VI and VII), and the process of protein refolding starts and is completed. The renatured protein is easily separated by recharging the nanoparticles or the protein by changing pH (step VIII), and finally, the nanoparticles are separated by centrifuging, and the renatured protein remains solution. The distinctive features of this approach are as follows: 1) the use of nanoparticles allows for up to 100% binding of the denatured protein molecules in a short period of time; 2) the process is non-specific, and does not require linkers – it is controlled only by electrostatics; 3) the alumina nanoparticles can be used repeatedly; 4) any protein with isoelectric point above 9.7 (most proteins) is a favorite candidate for this refolding procedure; 5) work with high protein concentrations is possible; 6) the renaturation process is quite general – even renaturation involving proteins with disulfide bonds is possible; 7) several protein types can be refolded at the same time.

**Figure 2:**
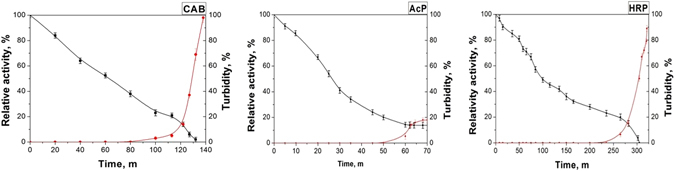
The effect of 8 M GdmCl denaturant on the relative activity and turbidity of a solution of CAB, AcP and HRP.

To prove the concept represented in the introduction, three enzymes were taken: carbonic anhydrase^26^, and the cancer-therapy enzymes acid phosphatase and horseradish peroxidase^27, 28^. To determine the optimal conditions for the denaturation and for the subsequent renaturation of the proteins, the behavior characteristics and aggregation stability of the molecules in a GdmCl solution have been studied. It was found that the optimal denaturant concentration is 8 M. The dependence of the relative activity and turbidity of the solution on denaturant exposure time was assessed. As seen in Fig. 2, after 80 minutes of treatment the aggregation of CAB molecules starts, and as a result, a substantial reduction in relative activity is observed. Similar curves were obtained for acid phosphatase and horseradish peroxidase.

**Figure 3:**
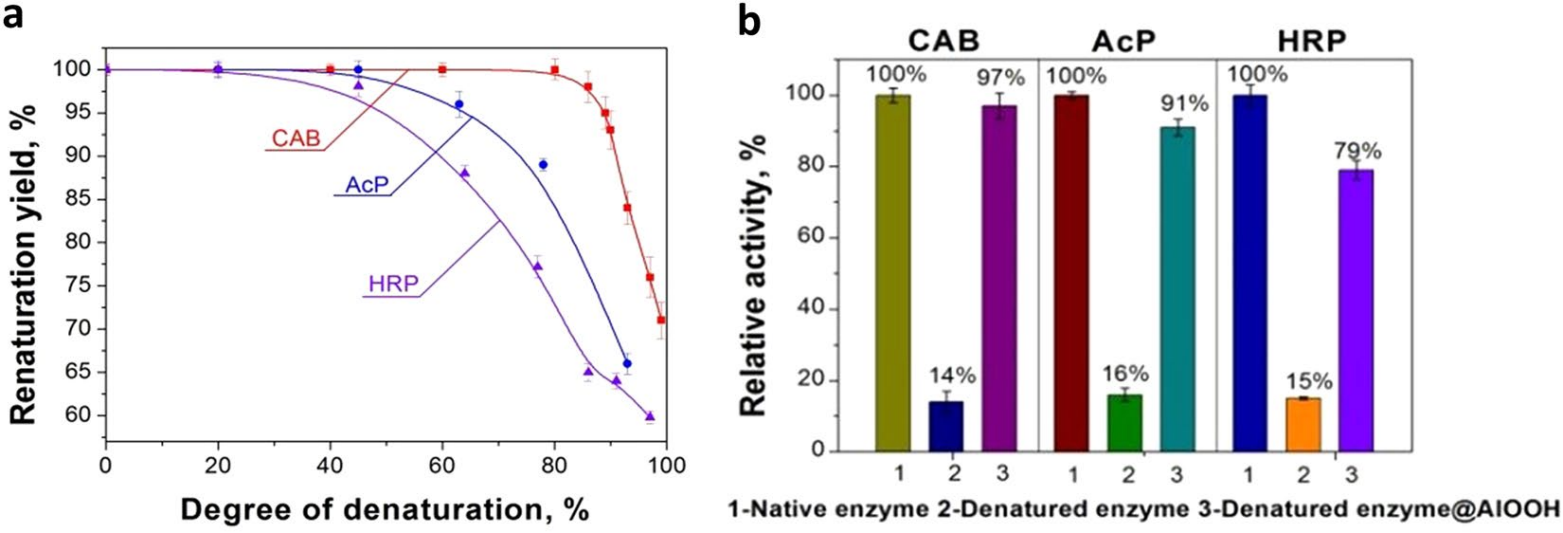
(**a**) Alumina NP-assisted renaturation yield as a function of degree of denaturation of CAB, AcP, HRP. (**b**) Renaturation yields of CAB, AcP and HRP molecules before separation from alumina NPs.

The evaluation of the renaturation took place in two stages. The first stage was to evaluate the activity of the enzyme still complexed with the alumina, and the second stage is the evaluation of the renaturing after separation from the alumina NPs. Regarding the first stage, the activity of the composite precipitated by centrifugation was compared to CAB molecules with different degrees of denaturation, and the results are shown in Fig. 3a (where the activity of native CAB precipitated with alumina NPs was taken as 100%). It is seen in the figure that we observed a 100% renaturation yield until a critical stage of denaturation degree of about 80%; aggregation could not be avoided beyond this stage. Similarly, the critical denaturation levels were determined for AcP, and for HRP, and were found to be 87% and 89%, respectively (see Fig. 3a). As seen in Fig. 3b, before the separation of the renatured molecules from the alumina NPs one observes high renaturation yields of 97% for CAB molecules and 91% for AcP, and a moderate renaturation yield of 79% for HRP. In this context we note that while CAB and AcP are metalloenzymes, HRP is a monomeric glycoprotein and has 4 disulfide bonds, which greatly complicates its renaturation; nevertheless, 79% is the maximal value reached so far for that enzyme^29^. We emphasize that the versatility and advantage of the method developed here, is also due to the ability of the high-yield refolded enzymes to be active while being linked with alumina NPs.

**Figure 4:**
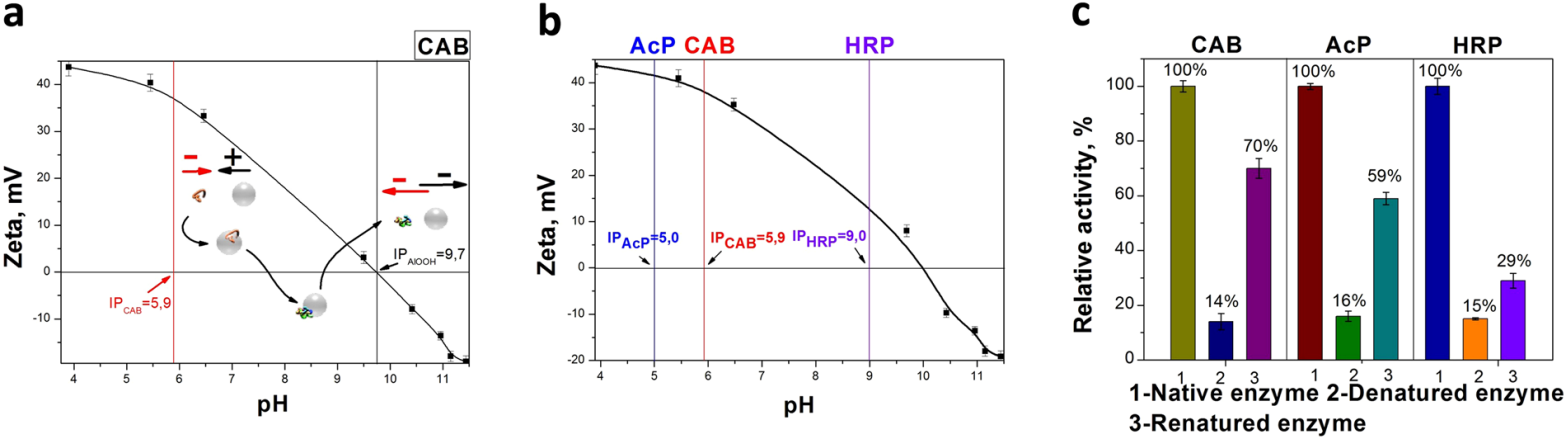
(**a**) The pH-dependence of the electrostatic interactions of CAB with the alumina NPs. (**b**) The zeta potentials of the alumina NPs and the isoelectric points of the three enzymes. (**c**) The renaturation yields of CAB, AcP and HRP after separation from the alumina NPs.

In order to facilitate the separation of the enzymes from the alumina NPs, further experiments were carried out with affecting the charge of the nanoparticles – under suitable pH conditions, the enzyme can be separated and purified by conventional centrifugation. The explanation and results of this step are given in Fig. 4: A representative scheme of the interactions of the enzymes – CAB in this example - with the alumina NPs is shown in Fig. 4a. Similarly, the conditions for separation were determined for AcP and HRP – see Fig. 4b. After the separation of the enzyme molecules, a comparison of activities of the renatured protein molecules and native ones was performed. As seen in Fig. 4c, the renaturation yield after the separation from alumina NPs is still appreciable for CAB and AcP – 70 and 59%, respectively, but low −29% - for HRP. The recovered alumina NPs can also be used for subsequent refoldings: the yield of CAB renaturation as a function of the recovery cycle of the same alumina gave following values: 70, 62, 55 and 40%.

**Figure 5:**
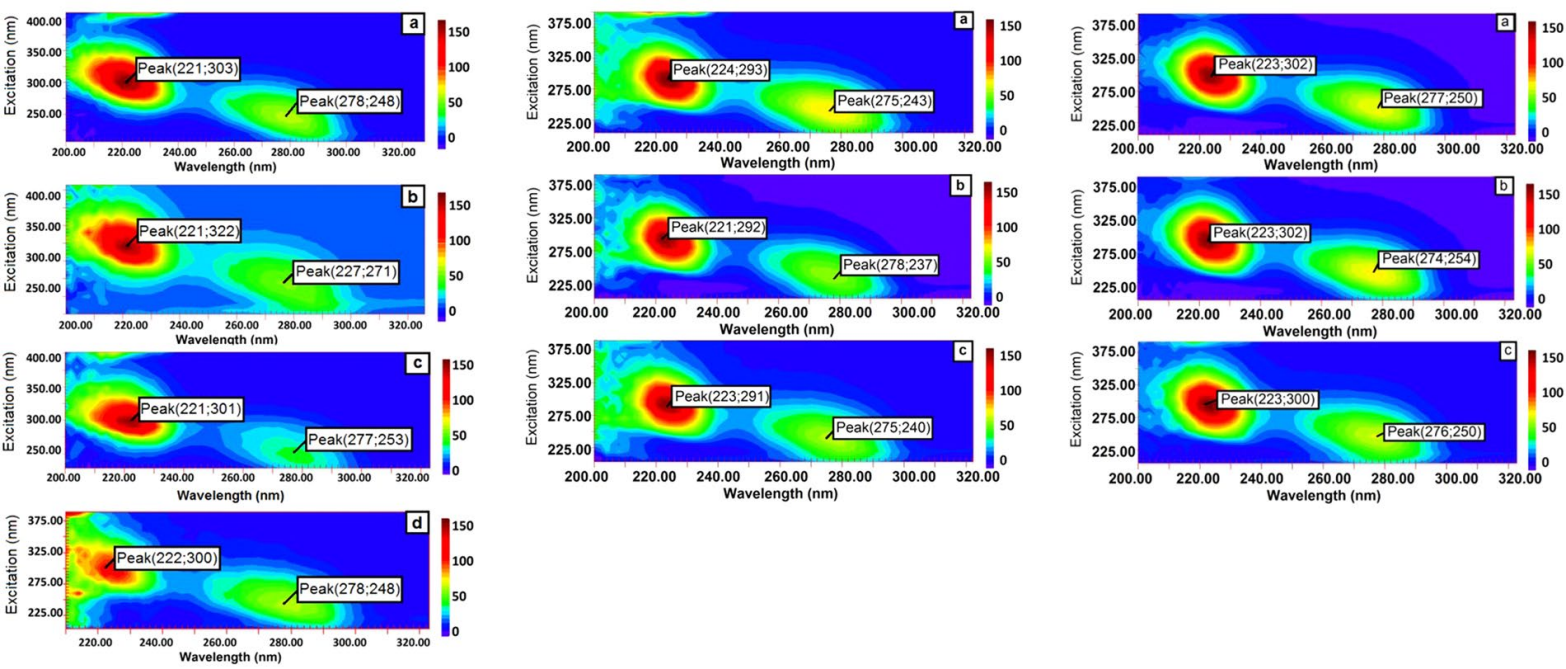
Synchronous fluorescence spectra of САВ, AcP, HRP native enzyme (**a**), denatured enzyme (**b**) renatured free enzyme (**c**) and renatured-complexed CAB (**d**).

Renaturation is also characterized by the regaining of native structure, as shown by synchronous fluorescence spectroscopy. Figure 5 shows the spectra of native, denatured, and renatured-complexed and renatured free CAB. As seen in the figure, the CAB molecules have two distinct excitation peaks at 221 and 278 nm with emission at 303 and 248 nm, respectively. After the denaturation, significant shifts of the main peak emission to 322 nm and of the minor peak to 271 nm are observed. After the alumina NP-assisted renaturation and subsequent separation, the CAB spectrum is almost completely reproduced. Similar results were obtained for HRP and AcP.

**Figure 6:**
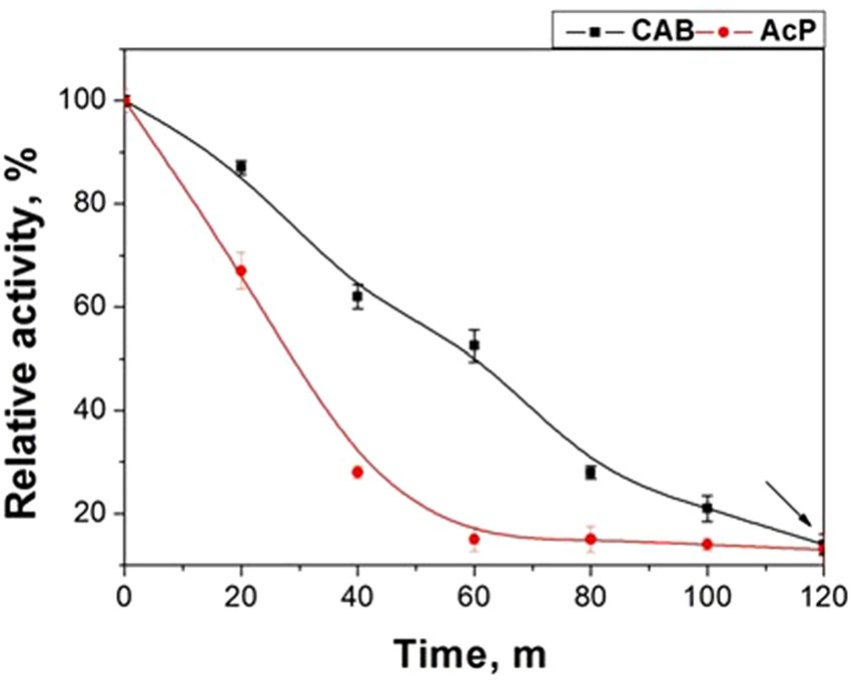
Effect of the presence of both CAB and AcP in 8 M GdmCl denaturant on the relative activity in solution.

The versatility of the proposed method has also been confirmed experimentally with renaturing two enzymes at the same time. Carbonic anhydrase and acid phosphatase were used simultaneously for this purpose. Alumina NPs were added after 120 minutes in 8 M GdmCl (see Fig. 6 and experimental part for details). At longer times of exposure to the denaturant, irreversible coagulation occurred and the renaturation process became impossible. Subsequently, renaturation yields before and after the separation of alumina NPs from the enzyme molecules were studied in accordance with the scheme shown in Fig. 1 (step VII or VIII). The results turned out to be in good agreement with those obtained previously (Fig. 2). The degree of renaturation for CAB and AcP amounted to 82 and 71%, while for free enzymes it was 59 and 48%, respectively.

A high yield, simple, non-specific method for protein renaturation has been developed, in which alumina NPs act at protein interface as effective chaperons for refolding. This study also demonstrates a new route for heterogenizing enzymes by the sol-gel methodology: Rather than entrapping the enzyme within the solid matrix, complexation with sol-gel nanoparticles is the new possibility. That complexation also solves the inherent problem of the sol-gel entrapment methodology, namely that the enzymatic reaction rates are slow because of the diffusion restrictions of the porous matrix – complexation solves this problem, because of the better accessibility of the enzyme. Of additional importance is that this work was carried out with alumina in the form of boehmite (as proven in previous reports^30^), an alumina form which is approved by FDA for the injection as the most common adjuvant. Taking into account that many therapeutic proteins are obtained from inactive inclusion bodies^31–33^, their parenteral administration can be safely provided with alumina-renatured enzyme complex. Alumina in this study was chosen due to high stability and zeta potential in the broad range of the pH and in our next studies we’ll also try to expand a developed method on other systems with similar behavior such as titania, zirconia and ferria.

## Experimental details

### Chemicals

Aluminum isopropoxide, carbonic anhydrase from bovine erythrocytes (CAB, cat. No. C3934), acid phosphatase from potato (AcP, cat. No.P3752), horseradish peroxidase (HRP, cat. No. 77332), p-nitrophenyl acetate (pNPA), p-nitrophenyl-phosphate (pNPP), 2, 2′-azino-bis (3-ethylbenzothiazoline-6-sulfonic acid) diammonium salt, guanidine hydrochloride was all obtained from Sigma-Aldrich. Tris-sulfate buffer was prepared from the respective solution with the desired volumes of 0.1 M NaOH. Glycine buffers were prepared from glycine solutions with volumes of 1.0 M HCl.

### Chemical denaturation of enzymes

Chemical denaturation studies were performed by denaturing 50 µl of CAB (12 500 U/mL) with 400 µL of 8 M Guanidine hydrochloride (GdmCl), or 50 µl of AcP (60 U/mL) in 400 µL of 8 M GdmCl, or 20 µl of HRP (750 U/mL) in 400 µL of 10 M GdmCl. Denaturation was stopped for CAB after 120 min, for AcP after 63 min and for HRP after 280 min, beyond which reversing the coagulation process becomes impossible.

### Renaturation

The enzyme-alumina NPs stage. Boehmite alumina NPs were prepared using a bio-friendly ultrasonic procedure as previously described^34, 35^. The mass content of alumina NPs is 2.5%. See Fig. 1S in the ESI Fig. 1S for additional characterization of the applied sol-gel alumina. For renaturation, 200 μL of the alumina NPs sol was added to a cuvette with the previously denatured enzyme, and the pH adjusted for electrostatic interaction of the protein molecules with the alumina, pH = 7.5 for CAB and AcP or pH = 9.4 for HRP. These pH values were achieved by the addition of 0.1 M NaOH. The protein – alumina complexed NPs were centrifuged at 6000 rpm for 5 minutes. The alumina NPs bound to the enzyme precipitate on the bottom, and the upper layer of the liquid with GdmCl was removed using a pipette. Afterwards, 500 μL of buffer solution (Tris-sulfate buffer for CAB, pH = 7.5; and glycine buffer for AcP and HRP, pH = 4.7) was added to the cuvette, and additional stages of removing the chemical denaturant were performed in a similar manner. A similar procedure was carried out with the native protein and the activity was compared. The complex can be stored after centrifugation as a final product and reused. Mixing. alumina NPs with the native enzymes without complexation does not lead to any change of activity. To carry out a test for the joint renaturation of two enzymes, CAB and AcP were used. To this end, 400 μL of alumina NPs was added to a cuvette with the previously denatured enzymes, 50 μL of CAB (12 500 U/mL) and 50 μL of ACP (60 U/mL), and as a result of electrostatic interaction the enzymes were bound to particles at pH = 7.5. Other operations were carried out in a way similar to that described above for individual enzymes. All experiments were performed at least three times.

### Separation of the renatured proteins from the alumina

To separate the nanoparticles from the renatured enzyme molecules, 0.1 M NaOH was added to the centrifuged alumina NPs until pH = 8, so that the renatured enzyme molecules and alumina NPs have the same charge, and after centrifugation, the particles precipitate on the bottom with the renatured molecules entering the solution. The alumina NPs remained in the cuvette can be used in subsequent renaturation processes. All experiments were performed at least three times.

**Figure 7:**
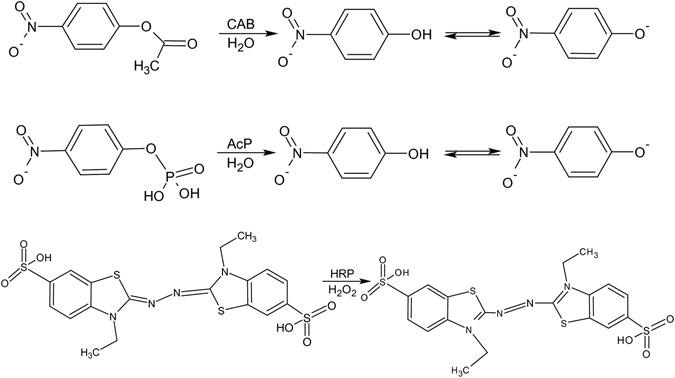
The enzyme-catalyzed reactions used in this study.

The enzymatic activity assays for native, denatured, and renatured enzymes (see Fig. 7 for reactions):

### CAB

Enzymatic activity of CAB was monitored using p-nitrophenyl acetate (pNPA) as a substrate. 63 μL of 52 mM pNPA in dry acetonitrile was added to 0.25 mg/mL CAB, 16 mM Tris-sulfate, pH 7.5 and 4.7 mM pNPA. After 10 s of mixing, the measurement of absorbance at 405 nm over a period of 600 sec was monitored. Hydrolysis of pNPA due to CAB results in phenolate ion (yellow color). An increase in absorbance at 405 nm is a measure of an increase in amount of the hydrolyzed acid product as the reaction proceeds. The rate of hydrolysis of pNPA is proportional to the amount of active protein in the solution. For reproducibility, at least three measurements under the same conditions were carried out. The natural hydrolysis of the substrate solution was measured as well and subtracted from activity of the samples.

### AcP

Enzymatic activity of 0.05 mg/mL АсР was monitored using 1.0 mL of 10.8 mM of the substrate (pNPP) in 2 mL of glycine–HCl buffer solution (pH 4.7), the enzymatic activity was measured by following the formation of p-nitrophenolate (pNPP) spectroscopically through absorption at 410 nm, at 37 °C.

### HRP

The enzymatic activities of native, denatured, and renatured HRP were determined using ABTS as a reducing substrate. The assay system was a mixture of 1.0 mL of 0.5 mM ABTS and 0.2 mL of 30% H_2_O_2_ in 2.0 mL of 50 mM glycine–HCl buffer (pH 4.7). The enzymatic activity was measured spectrophotometrically as a rise of absorbance of the oxidized ABTS at 425 nm.

### Characterization techniques

The spectral analysis of enzymatic activity was carried out using an HP 8453 Diode Array spectrophotometer. Dynamic light scattering measurements were carried out using a Compact Z Photocor Instrument. Synchronous fluorescence spectra were recorded with a Cary Eclipse fluorimeter. Determination of viscosity was carried out using a Fungilab EXPERT viscometer.

The crystalline phase and crystallinity of the samples were investigated by X-ray diffraction (Bruker D8 Advance) using Cu-Kα radiation (λ = 1.54 Å); the samples were scanned at 2 $\theta$ at a rate of 0.5 degrees per minute. To analyze the samples using high-resolution scanning electron microscopy (SEM), the obtained ground xerogel was deposited on a metal tip and investigated without additional spraying using a Magellan 400 L ultra-high resolution electron microscopy (TEM) were obtained by dispersing a small probe in ethanol to form a homogeneous suspension. Then, a suspension drop was coated on a copper mesh covered with carbon for a TEM analysis (FEI TECNAL G2 F20, at an operating voltage of 200 kV).

## Electronic supplementary material

Supplementary Information
