# Supplementary material for: Alumina nanoparticle-assisted enzyme refolding: A versatile methodology for proteins renaturation
Source: Sci Rep. 2017 May 3;7:1458. doi: 10.1038/s41598-017-01436-6 (PMC5431136; doi:10.1038/s41598-017-01436-6)
Supplement: Supplementary file 1 — Supplementary Information [file 41598_2017_1436_MOESM1_ESM.pdf]

Alumina nanoparticle-assisted enzyme refolding: A versatile methodology for proteins renaturation

Katerina V. Volodina[1], David Avnir[2] and Vladimir V. Vinogradov[1*]

[1]ITMO University, Laboratory of Solution Chemistry of Advanced Materials and Technologies,

Lomonosova St. 9, 191002, St. Petersburg, Russian Federation

E-mail: vinogradov@scamt.ru

[2] Institute of Chemistry and the Center for Nanoscience and Nanotechnology, the Hebrew University of Jerusalem, Jerusalem 9190402, Israel

Electronic Supplementary Information

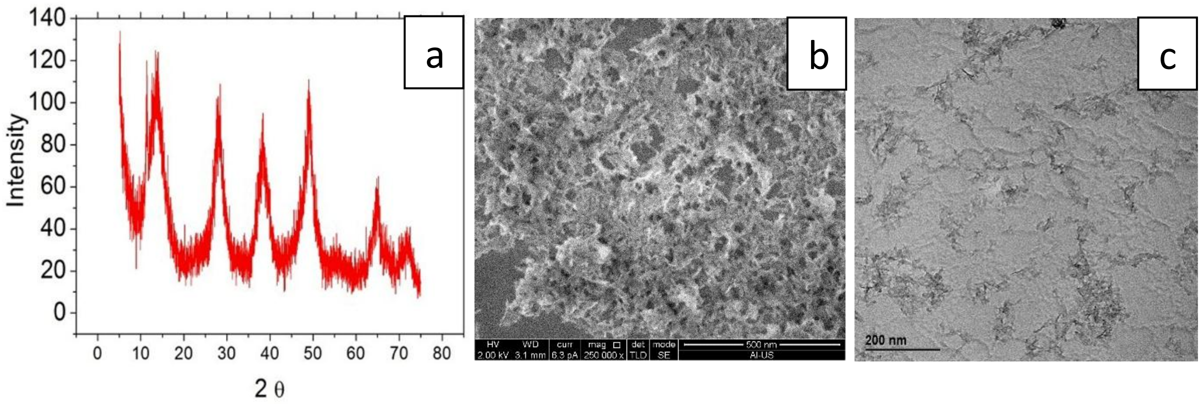

Figure 1S. Characterization of alumina sol – gel matrix used in the study

The positions of the maxima in the XRD spectrum (a) correspond to a structure of boehmite. An analysis of the size of the crystallites - carried out with the Scherrer equation - indicates an average particle size of 3–4 nm. These results are in a good agreement with SEM (b) and TEM (c) images revealing rod-like nanoparticles 3-10 nm in size.
